# Cell Death Induced on Cell Cultures and Nude Mouse Skin by Non-Thermal, Nanosecond-Pulsed Generated Plasma

**DOI:** 10.1371/journal.pone.0083001

**Published:** 2013-12-16

**Authors:** Arnaud Duval, Ilya Marinov, Guilhem Bousquet, Guillaume Gapihan, Svetlana M. Starikovskaia, Antoine Rousseau, Anne Janin

**Affiliations:** 1 Inserm, U 728, Paris, France; 2 Université Paris Diderot, Sorbonne Paris Cité, Laboratoire de Pathologie, UMR-S 728, Paris, France; 3 AP-HP (Assistance Publique-Hôpitaux de Paris), Hôpital Saint-Louis, Department of Pathology, Paris, France; 4 Laboratoire de physique des plasmas, Ecole polytechnique, UPMC, Université Paris Sud 11, CNRS, Palaiseau, France; National Research Council, Italy

## Abstract

Non-thermal plasmas are gaseous mixtures of molecules, radicals, and excited species with a small proportion of ions and energetic electrons. Non-thermal plasmas can be generated with any high electro-magnetic field. We studied here the pathological effects, and in particular cell death, induced by nanosecond-pulsed high voltage generated plasmas homogeneously applied on cell cultures and nude mouse skin. In vitro, Jurkat cells and HMEC exhibited apoptosis and necrosis, in dose-dependent manner. In vivo, on nude mouse skin, cell death occurred for doses above 113 J/cm^2^ for the epidermis, 281 J/cm^2^ for the dermis, and 394 J/cm^2^ for the hypodermis. Using electron microscopy, we characterized apoptosis for low doses and necrosis for high doses. We demonstrated that these effects were not related to thermal, photonic or pH variations, and were due to the production of free radicals. The ability of cold plasmas to generate apoptosis on cells in suspension and, without any sensitizer, on precise skin areas, opens new fields of application in dermatology for extracorporeal blood cell treatment and the eradication of superficial skin lesions.

## Introduction

Physical gas plasma is a well-known phenomenon generated by intense electromagnetic fields. It is a gas that has become ionized because considerable energy has been transferred to it. So it is the fourth state of matter after solid, liquid and conventional gas. It may occur as flashes in a storm, argon electric light, fire/lightning sparks or sun corona [1]. A plasma is a mixture of free radicals, ionized molecules, reactive oxygen species, and light, and can be thermal or non-thermal. In non-thermal plasmas (NTP), the gas is partially ionized and their temperature remains close to ambient temperature, which makes them suitable for biological applications. So far, current medical applications are scarce and mainly concern sterilisation [2]. 

However numerous potential medical applications have been suggested by different studies : blood coagulation [3-6], wound healing [7–9], treatment of cancer [10–15]; infection [16], and skin rejuvenation [17]. Preclinical studies of Nanosecond-Pulsed Plasma (NPP) for dermatological applications are however lacking. We chose NPP because its homogeneity in space and well-controlled energy per pulse makes it preferable for skin treatment. We investigated the pathological effects of NPP in vitro and in vivo to explore the therapeutic possibilities of this type of plasma in preclinical models in the field of dermatology.

## Results

### Plasma characteristics

**Figure 1:**
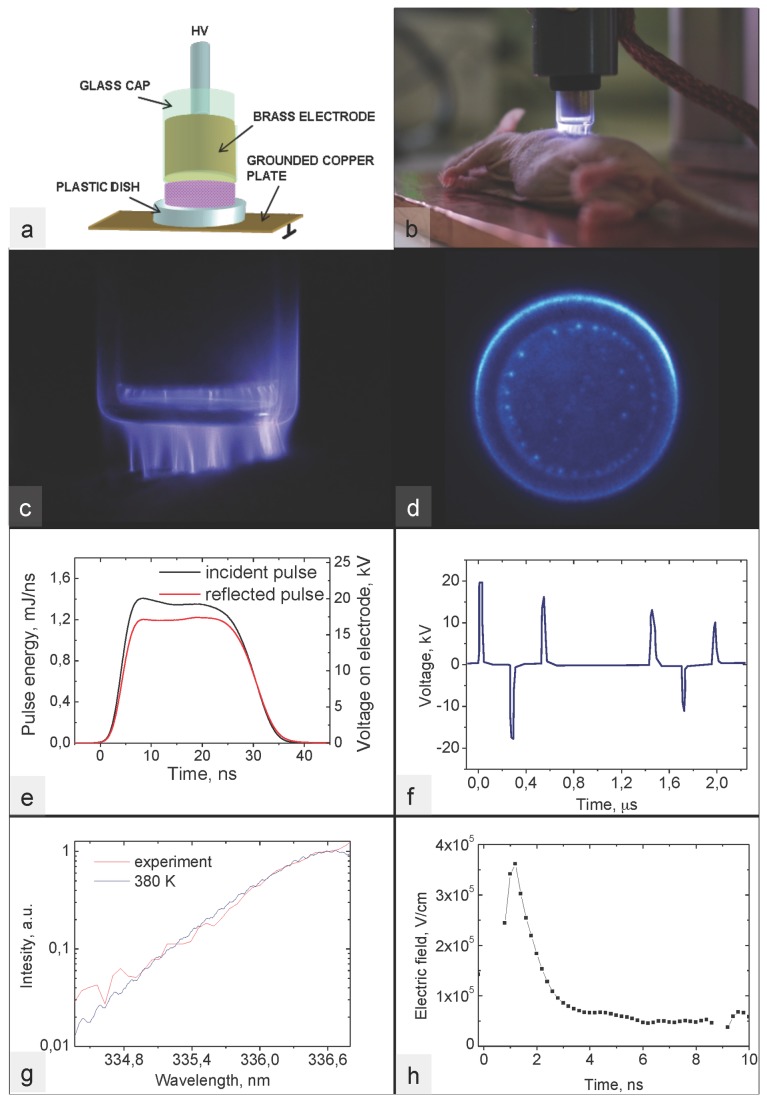
Device description, plasma characteristics. Device description. a: A high voltage current (HV) is conducted to a brass electrode insulated with a glass cap. The plastic dish is placed between the capped electrode and the ground plate. b: Image of the device during an in vivo experiment. Plasma characteristics. c: Profile image of the discharge. d: iCCD imaging of a discharge. Single shot. Camera gate is 40 ns. e: Power of incident and reflected HV pulses measured by back current shunt. f: Back current shunt signal. Six successive pulses of alternate polarity with maximum amplitude of +9.5 kV, -8.5 kV, +7.5 kV, +5.5 kV, 4.7 kV, 4 kV. g: Experimental and calculated optical emission spectroscopy of 337.1 nm band of N_2_ 2^+^ system. h: Electric field profile during first positive pulse.

Our device is depicted in Figures 1a, 1b, 1c and 1d. The characteristics of the plasma, namely, discharge homogeneity (Figure 1d), pulse energy (Figure 1e), voltage pulses (Figure 1f), temperature (Figure 1g) and electric field (Figure 1h), are presented below.

We evaluated the homogeneity of the plasma discharge and Figure 1.d shows an intensified Charge-Coupled Device (iCCD) camera image of the discharge integrated over 50 ns at 500 Hz of High Voltage (HV) pulse repetition rate. It can be noted that the discharge has a uniform overall pattern with a slight increase in intensity at the electrode and dielectric edges. Several bright spots can be observed on the electrode surface, which are due to its surface roughness.

We evaluated the energy necessary to generate the plasma discharge by comparing the energy of the incident pulse, corresponding to HV pulse travelling from the generator to the discharge device, and the reflected pulse coming back from the plasma device (Figure 1e). We obtained a series of 6 successive pulses of alternate polarity (Figure 1f) with an energy input in the plasma of 3.8 mJ, 3.2 mJ, 3.4 mJ, 2.8 mJ, 2.2 mJ and 2.4 mJ respectively. The overall energy deposited in the plasma for one HV pulse was about 18 mJ, corresponding to 10mJ/cm^2^ for an electrode diameter of 17 mm.

The temperature was deduced from the optical emission spectrum shown (Figure 1.g), accumulated during the first positive pulse and averaged over 100 shots. It gives 380 K for the gas temperature in the plasma during application of the voltage pulse. This temperature occurs only during the 30 ns of the plasma discharge, which amounts to less than 0.01% of the treatment time. Hence the time-averaged gas temperature remains close to ambient temperature.

Concerning the electric field, Figure 1.e shows a narrow peak of about 3.7x10^5^ V/cm, corresponding to the rising slope of the pulse voltage applied. Then a fast decrease of the electric field with a constant voltage during the pulse plateau is observed.

### In vitro characterisation of cell death on HMEC and Jurkat cells

**Figure 2:**
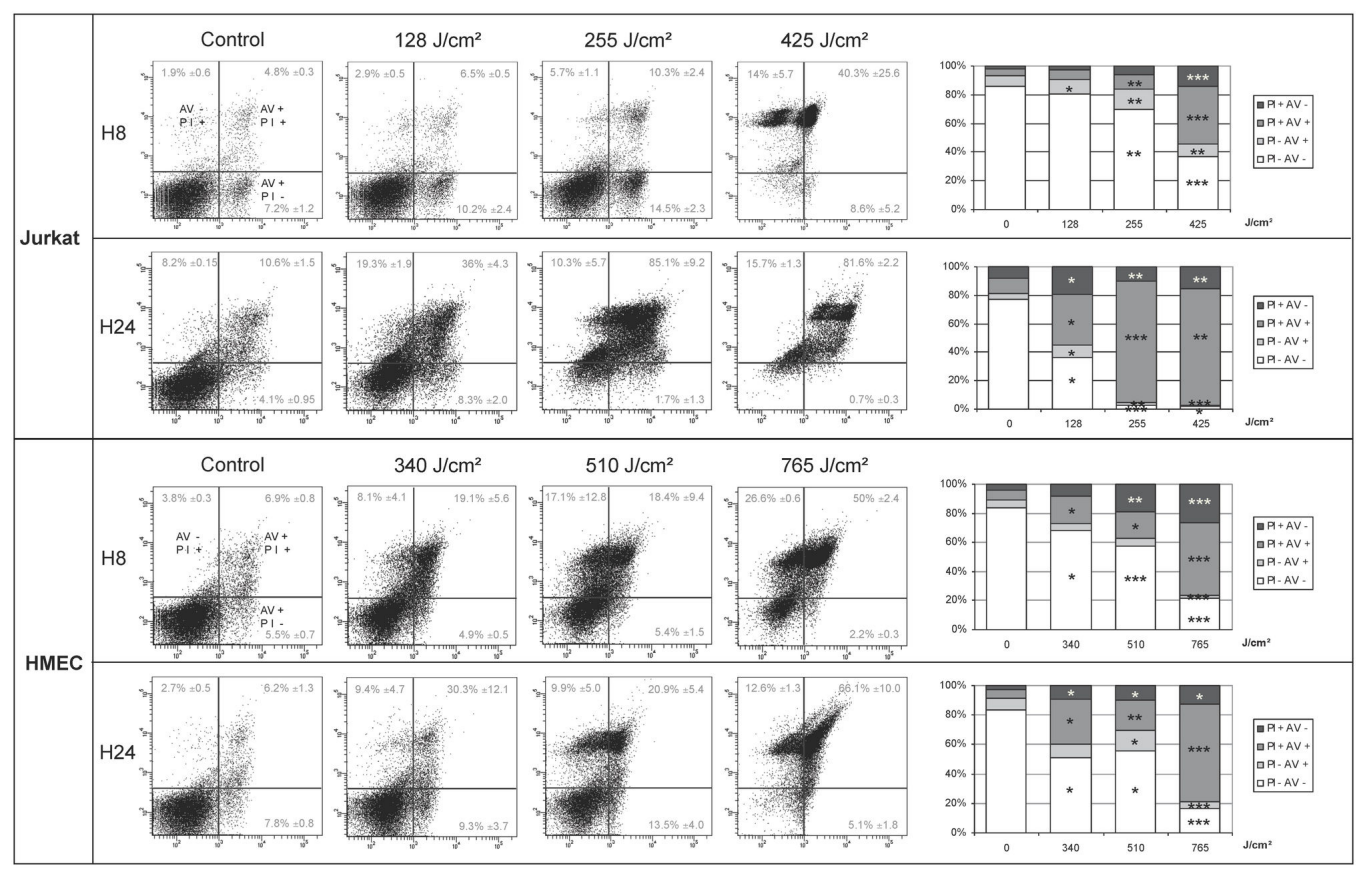
Annexin V / PI assay on HMEC and Jurkat cells 8 and 24 Hours after plasma treatment. Cells were treated for 30 to 120 sec and from 75 to 900 Hz frequency. The administered dose is expressed in J/cm^2^. Flow Cytometry data are analysed on a four quadrant dot plot gated on a region including cells and debris. Propidium iodide (PI) signal is detected in the Phycoerythrine channel, on the Y axis. FITC-labelled Annexin V signal is detected on the X axis. Non-apoptotic, non-necrotic, living cells are located in the lower left quadrant. Results are expressed as median +/- standard deviation. On the right, for each experimental time, a histogram including statistical analyses summarizes cytometry data. Statistical significance: * means p<0.05 when compared with the control; ** means p<0.05 when compared with the immediately lower dose, *** means p<0.05 when compared with the control and the immediately lower dose. On Jurkat cells, 8 hours after treatment, the type of cell death is mainly apoptosis, up to 255 J/cm^2^. Above this dose, the type of cell death is mainly necrosis. At H24, the pattern is non-specific, consistent with late apoptosis or necrosis. Overall the data are consistent with the hypothesis that NPP induces apoptosis on Jurkat cells up to the dose of 255 J/cm^2^. On HMEC, 24 hours after treatment, at 510 J/cm^2^, there is an increase of Annexin V-only positive cells consistent with apoptosis. Otherwise the pattern is mainly necrotic. On HMEC and Jurkat cells, there is a dose effect. To reach a given percentage of dead cells, HMEC cultures need higher doses than Jurkat cells.

Flow cytometry data were analysed on a four-quadrant dot-plot gated on a region including cells and debris. A propidium iodide (PI) signal was detected in the Phycoerythrine channel, on the Y axis. A FITC-labelled Annexin V signal was observed on the X axis. Non-apoptotic, non-necrotic, living cells were located in the lower left quadrant. The apoptotic pattern is defined as follows: in early-stage apoptosis, cells are labelled with Annexin V only. Later, since the plasmic membrane integrity is altered, they become positive for both Annexin V and PI. The necrotic pattern is defined as follows: cells become positive for both Annexin V and PI at the same time because of the alteration of the plasmic membrane. There is no solely Annexin V- positive cell [18]. Data are shown in Figure 2. Cells were treated for 30 to 120 sec using 75 to 900 Hz frequency. The dose administered was expressed in J/cm^2^.

On Jurkat cells, 8 hours after treatment, the pattern is mainly apoptotic up to 255 J/cm^2^. Above this dose, the pattern is necrotic. At H24, the pattern is non-specific, consistent with late apoptosis or necrosis. Overall the data are consistent with the hypothesis that NPP induces apoptosis on Jurkat cells up to the dose of 255 J/cm^2^.

On HMEC, 8 hours after treatment, for doses higher than 340 J/cm^2^, the pattern was not specific, consistent with late apoptosis or necrosis. 24 hours after treatment, at 510 J/cm^2^ we observed an early increase in Annexin V-only positive cells, consistent with apoptosis. Otherwise the pattern was mainly necrotic. HMEC therefore needs a higher dose of plasma to induce cell death. NPP mainly induces necrosis, and apoptosis to a lesser extent.

On HMEC and Jurkat, there is a dose-effect. To reach a given percentage of dead cells, HMEC cultures need higher doses than Jurkat cells.

### NPP action mechanism

The type of the plasma UV emission was studied with a spectrophotometer. Electronic transitions of the molecules NO, OH and N2 were detected, corresponding to UV A, B and C respectively. The intensity of the UV emission was evaluated with a UV-meter designed for clinical applications. The absolute intensity was lower than the detection threshold.

pH measurements were performed in triplicate in 300 µl of plasma-treated medium. The pH of the Jurkat medium remained at 7.2 up to the highest dose of plasma. A dose three times higher than the highest treatment dose used for cells caused a decrease in pH to 6.1. Under plasma treatment the HMEC culture medium pH decreased from 8.6 to 7.7 (maximum plasma dose used for cells).

On the surface of mouse skin, the temperature increase was less than 1°C.

**Figure 3:**
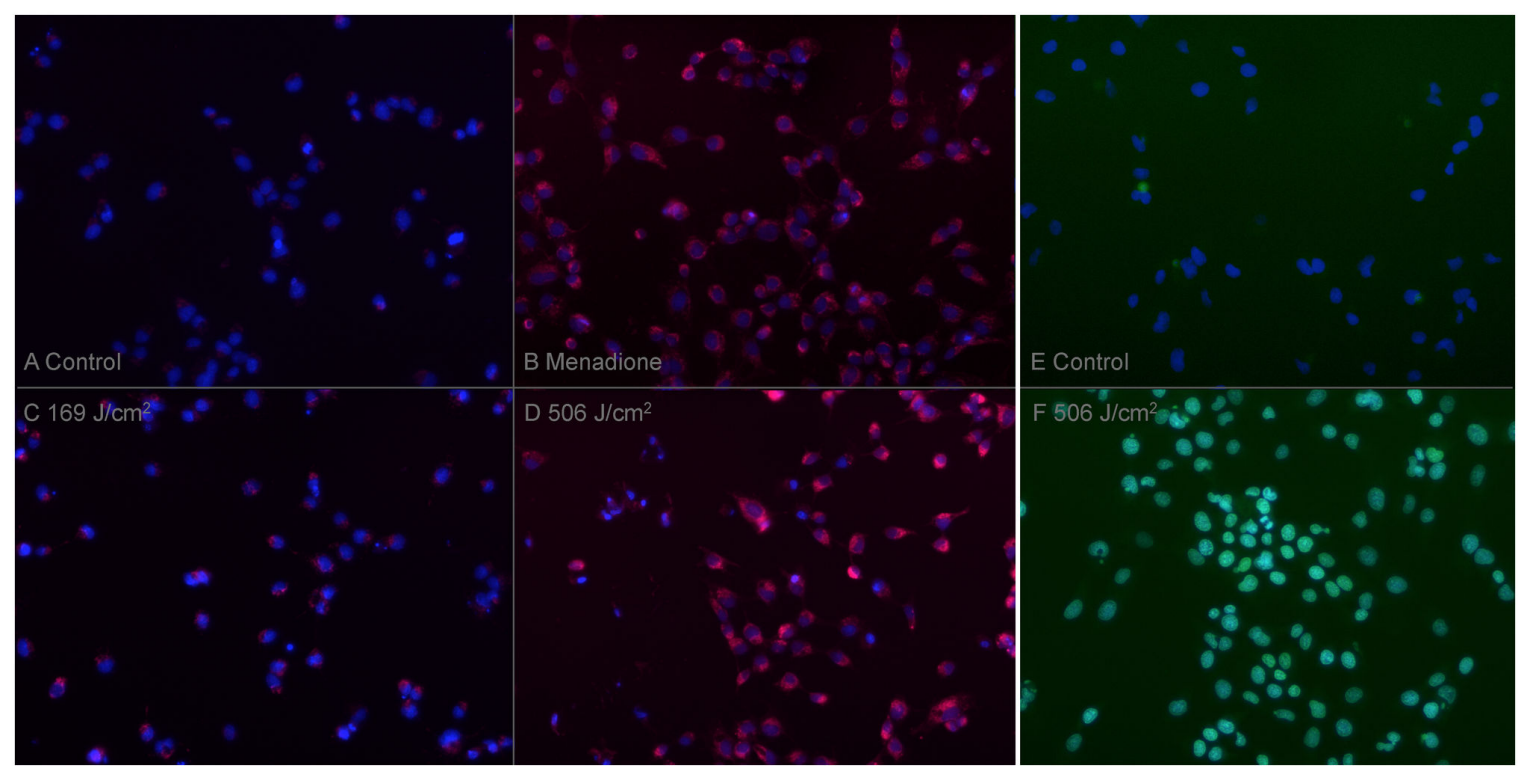
Oxydative stress assay (A-D), Electroporation assay (E-F). For both tests, nucleuses were labelled with NucBlue, images were taken with the same parameters as for the negative control. Oxydative stress assay: Cells were incubated one hour after treatment by plasma or menadione 100µM with Cellrox deep green, an intracytoplasmic fluorescent marker of oxidative stress. A, negative control, almost no fluorescence is apparent. B, Menadione, a strong cytoplasmic red fluorescence is present. C and D, medium and intense fluorescence depending on the plasma dose, 169 and 508 J/cm^2^ respectively. Electroporation assay: Cells were incubated just before plasma treatment with Sytox green, a fluorescent marker of membrane impairment. E, negative control, almost no fluorescence is visible. F, high-dose plasma treatment (506 J/cm^2^), a strong green fluorescence is present.

Intracellular oxidative stress was evaluated on HMEC with an intracytoplasmic fluorescent marker (Cellrox® Red). Red fluorescence appeared for doses above 169 J/cm^2^ and increased up to the maximum dose of 506 J/cm^2^ (Figure 3).

Electroporation was tested on HMEC with a fluorescent DNA stain (Sytox green®) which penetrates only the cells with membrane damage. Strong fluorescence appeared for high doses (506 J/cm^2^). No fluorescence could be seen for lower doses (Figure 3).

### Pathological effects of NPP on nude mouse skin

**Figure 4:**
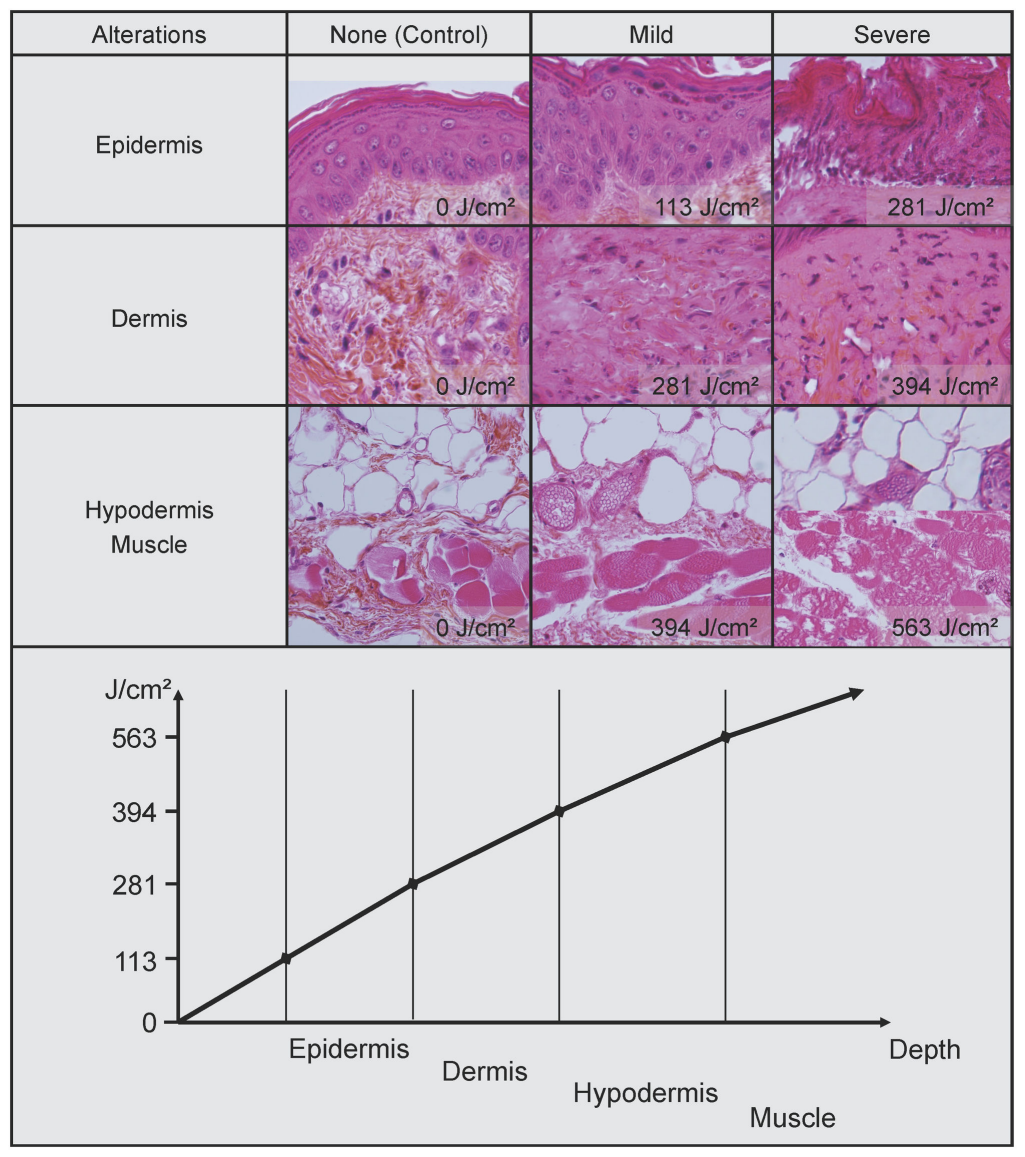
Hematoxylin Eosin sections of nude mouse skin after plasma treatment. Epidermis, dermis, hypodermis and muscle are affected for doses higher than 113 J/cm^2^, 281 J/cm^2^ and 394 J/cm^2^ respectively. In the diagram in the lower quadrant, the doses necessary to produce mild lesions for each layer of the skin are shown.

A dose-effect was found clinically and microscopically. 24 hours after treatment, clinical abnormalities were present for doses higher than 300Hz lasting 30 seconds (169 J/cm^2^) and increased gradually with the dose. For high doses, above 700Hz for 30 sec (394 J/cm^2^), the skin was necrotic and subcutaneous tissues were damaged. Microscope observation of HE sections of nude mouse skin 24 hours after treatment showed similar abnormalities whether it was the time or the frequency that varied. Therefore, each treatment is expressed as an energy emitted (J/cm^2^) rather than as a frequency with a duration of treatment (Figure 4). No abnormality could be seen below 113 J/cm^2^. At 113 J/cm^2^ significant epidermal abnormalities were present. The delineation of individual cells was partly lost, the cytoplasms were strongly eosinophilic and the nucleuses were often picnotic. There was no visible abnormality below the epidermis. At 281 J/cm^2^, the epidermis was severely damaged and considerable cell detachment was present. The dermis was oedematous. At 394J/cm^2^, the epidermis and the dermis were severely affected with no identifiable collagen fibre. The hypodermis and the muscle had oedema and vasodilatation. At 563 J/cm^2^, the hypodermis and the muscle layer were severely damaged.

**Figure 5:**
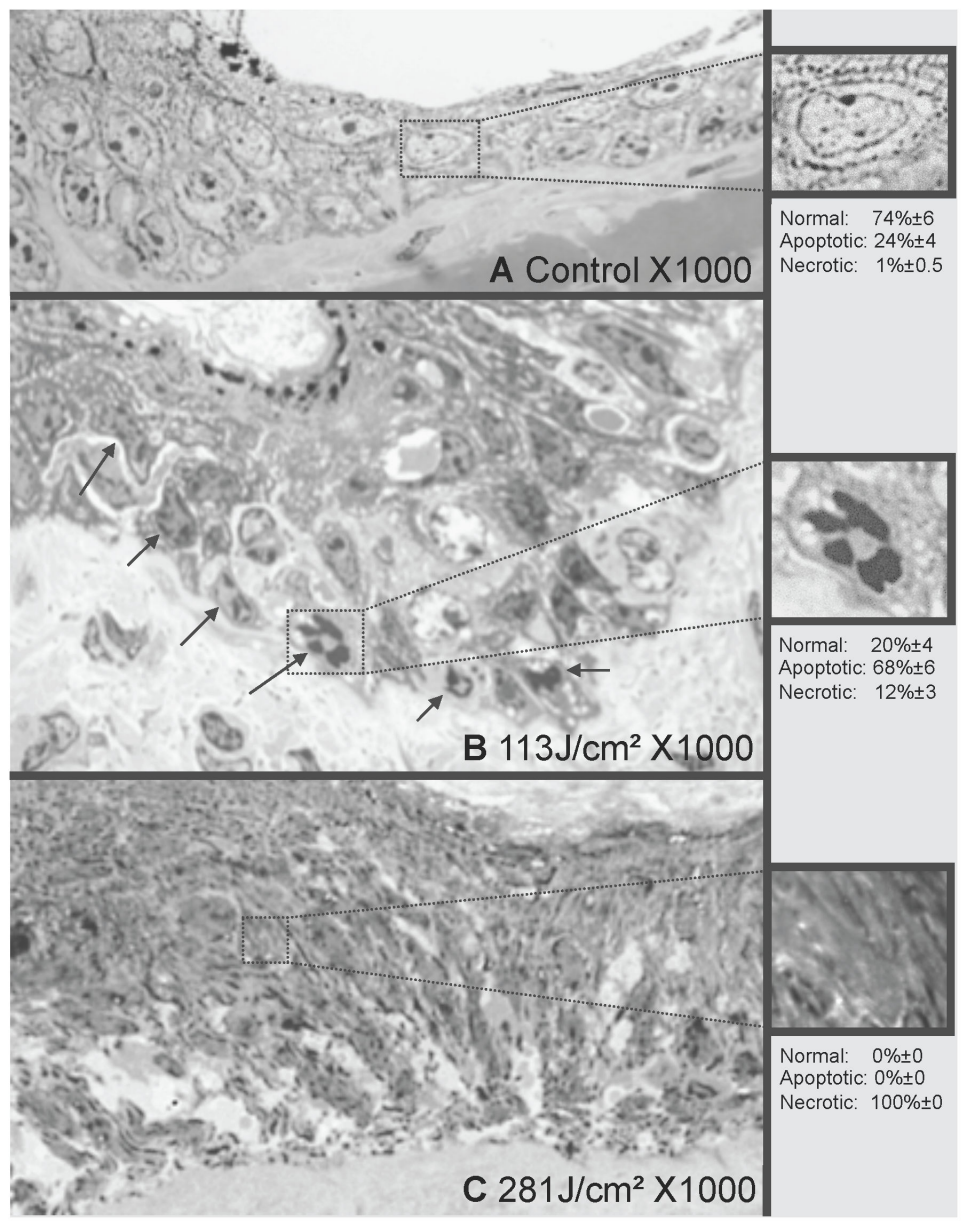
Electron microscopy of nude mouse epidermis after plasma treatment. For each sample, on five different fields, 100 cells were counted and classified as normal, necrotic or apoptotic. Results were expressed as median +/- standard error. Representative pictures are shown: A: Normal skin. B: 113 J/cm^2^. Numerous apoptotic keratinocytes are present, presenting highly condensed chromatin. C: 281 J/cm^2^. The epidermis is completely disrupted, no cell is distinguishable. On the control samples, the normal, apoptotic and necrotic cell percentages were 74%±6, 24%±4 and 1% ±0.5 respectively. At the doses of 113 J/cm, the normal, apoptotic and necrotic cell percentages were 20%±4, 68%±6, 12%±3 respectively. These results were statistically different from the results of normal samples (p<0.001). At the dose of 281J/cm^2^ normal cells 0%±0, apoptotic cells 0%±0, necrotic cells 100%±0. These results were statistically different from the results of samples treated at 113J/cm^2^ (p<0.0001).

Cell death was characterized on electronic transmission microscopy (Figure 5). The results were: i) on the control samples: normal cells 74%±6, apoptotic cells 24%±4, necrotic cells 1% ±0.5; ii) at the dose of 113 J/cm^2^: normal cells 20%±4, apoptotic cells 68%±6, necrotic cells 12%±3; These results were statistically different from the results of normal samples (p<0.001); iii) at the dose of281J/cm²: normal cells 0%±0, apoptotic cells 0%±0, necrotic cells 100%±0.

These results were statistically different from the results of samples treated at 113J/cm^2^ (p<0.0001).

Representative images are shown in the Figure 5. No significant abnormality was noticeable on the control (A). At the dose of 113 J/cm^2^ (B), significant vacuolisation of epidermal cells was found, associated with marked chromatin condensation consistent with apoptosis. At 281 J/cm^2^ (C), almost no structure remained distinguishable in the epidermis, consistent with necrosis.

## Discussion

In this work, we showed that the NPP produced by our device is homogeneous and induces cell death in vitro on cell cultures and in vivo on nude mouse skin. There is a dose-effect, since the higher the dose, the more numerous are the cells killed and the deeper are the tissues affected. Induced cell death is mainly apoptotic for medium doses, necrotic for high doses, and it is related first to the production of reactive oxygen species and secondly to electroporation at high doses.

NTP is a physical phenomenon and its possible applications in medicine are beginning to be explored. NTP is a cold, weakly ionized gas which consists to a large extent of charged particles such as ions, electrons, and free radicals. It emits little UV radiation and light (optical and infrared). It is generated by high electromagnetic fields; however the discharge current is kept low by inserting a dielectric insulator (usually plastic or gas) between the two electrodes and/or by using a nanosecond voltage pulse. We used a volume barrier discharge device in which the target is located between the two electrodes. 

We first wanted to know whether our device was suitable for skin applications. Measurement of plasma gas temperature yielded 380 K, a temperature usually lethal for living cells. However, the consequences of this increase in gas temperature are limited by the very short duration of the discharge (less than 0.01% of the treatment time) and by a moderate repetition rate of the nanosecond pulses (typically less than 900 Hz). So the overall energy contributing to heating is sufficiently moderate to apply this type of discharge directly to cell cultures or living tissues. If we consider skin applications, it is crucial to be able to treat a given area homogeneously. With iCCD capture at 1 second, we confirmed that plasma is delivered homogeneously over the whole treated area, one of the main interests of nanosecond NTP. 

We performed in vitro experiments on Jurkat cells and HMEC, and also in vivo experiments on nude mouse skin. To explore the whole spectrum of the effects of NPP, we applied a wide range of doses. By dose, we refer to the energy necessary to generate the plasma discharge, and not the energy received by the target. We focused our interest on the type of induced cell death and on the pathological effects on tissues. We obtained similar results in vitro and in vivo. In vitro, flow cytometry results on Jurkat cells showed that there was no effect at very low doses, an apoptotic pattern for medium doses, and a purely necrotic pattern for high doses. In each instance there was a dose-effect. On HMEC, the doses required were higher. Thus, attached cells seem to be more resistant to apoptosis. This may be due to the anti-apoptotic stimulation mediated by their cell-cell and cell-matrix junctions [19]. In vivo, on hematoxylin-eosin sections, significant lesions appeared for doses above 113 J/cm^2^ for the epidermis, 281 J/cm^2^ for the dermis, and 394 J/cm^2^ for the hypodermis and muscle. Thus, the higher the dose, the deeper is the effect in the tissue. On the epidermis, pathological analysis on ultra-thin sections was consistent with apoptosis for medium doses (113 J/cm^2^) and necrosis for higher doses (281 J/cm^2^). 

We investigated physical effects induced by the plasma that could be involved in the biological effect described above. We showed that this effect was not related to temperature elevation, UV emission or pH variation. We found that reactive oxygen species were present in significant concentrations in the cells. Oxidative stress has been known for a long time to be able to induce cell death. Consistently with previous publications on sinusoidal current-induced plasma, this seems to be the main physical effect by which plasma induces apoptosis and necrosis, but it may not be the only one. Electric breakdown of air at atmospheric pressure requires at least 30kV/cm to maintain the discharge. A high-voltage electric field is known to induce pore formation in cell and intracellular membranes [20] and this fact is used in conventional electroporation for cell transfection or drug delivery. Short nanosecond high-voltage pulses have been shown to affect the membranes of intracellular organelles, resulting in membrane permeabilization [21], intracellular calcium release [22], DNA damage [23] and apoptotic behavior [24]. We tested the membrane permeabilization of HMEC during plasma treatment. Although the evaluation of the size of the pores detected was not possible, we showed that significant membrane permeabilization occurs at high doses, 506 J/cm^2^.

NPP appears to induce apoptosis and necrosis on skin by generating free radicals, targeting a particular field, even if this may not be its sole mechanism of action. This mechanism of action recalls photodynamic therapy (PDT). PDT is based on the administration of a photosensitive agent (photosensitizer) activated by local irradiation with light at an appropriate wavelength. When the photosensitizer is activated, it induces the production of free radicals and subsequent cell death [25]. On skin, there is no evidence for tumor specificity of this technique [26,27]. There are several drawbacks with this therapy. First the photosensitizer is very costly (5-aminolevulinic acid, in France 207 euros/2 grams, Metvixia^®^). Second, the effect is superficial, and third it is difficult to accurately modulate the intensity of the effect. In addition the procedure is quite long since a time lapse of 3 hours is necessary between the application of the cream and the light treatment. Because no sensitizer is necessary, because it is possible to modulate the depth and the intensity of the treatment, and because the treatment time is very short, NPP might be an interesting alternative to PDT, especially to treat superficial skin cancers such as superficial basal cell carcinoma.

NCPI induces apoptosis of Jurkat cells in suspension. Jurkat cells are an immortalized line of T lymphocytes used to study T-cell lymphomas. Cutaneous T-cell lymphomas (CTCL) can be treated with topic treatments, systemic chemotherapy and/or extracorporeal photopheresis. The mechanism of action of extracorporeal photopheresis is still not completely understood. However the key effect seems to be that it induces apoptosis of circulating lymphoma cells. These apoptotic cells are re-infused into the patient and generate an anti-tumorogenic immune response [28,29]. The beneficial effects of plasmapheresis are usually temporary. NPP might be an additional tool to extracorporeal photopheresis, as an adjuvant or an alternative.

The ability of cold plasmas to generate apoptosis without any sensitizer on cells in suspension and on precise skin areas opens new fields of application in dermatology: cancer treatment, extracorporeal blood cell treatment and eradication of superficial skin lesions.

## Materials and Methods

### Reagents

Fluorescein isothiocyanate (FITC)-conjugated Annexin V, Propidium iodide (PI), Cellrox Red® (fluorescent marker of intracytoplasmic oxidative stress), and Sytox Green® (marker for impaired plasmic membrane) were obtained from Invitrogen/Paisley/UK; Menadione (oxidative stress inducer) from Sigma-Aldrich/St.Louis/USA. Trypsin-EthylenDiamineTetrAcétic (0.05%/0.02%) from PAA/Piscataway/USA and Trypsin-Neutralizing-solution from CELL/SanDiego/USA.

### Cell cultures

Jurkat and HMEC are immortalized cell lines of human lymphocytes and microvascular endothelial cells respectively [30,31]. They were obtained from ATCC/Virginia/USA. Cells were grown in non-coated T25 culture flasks. The culture medium was made up of RPMI with 10% foetal-calf- serum for Jurkat cells, and of MCDB131 with 10% foetal-calf-serum, 10ng/mL epidermal-growth-factor and 1µg/mL hydrocortisone for HMEC. Experiments were performed in 24-well plates seeded with 60000 cells 48 hours previously. 

### NTP technique

A strong electric field is generated between two electrodes to ionize the ambient air. To avoid any deleterious electrical discharge, the current used is nanosecond-pulsed and a glass cap is placed in front of one of the electrodes. A commercial high-voltage nanosecond pulse generator based on ultra-fast solid-state switches is used. It is produced by FID GmbH. An applied model (FPG 10) delivers monopolar positive pulses in single-shot regime or in repetitive mode (maximum frequency 1kHz), of 30 ns duration with 5 ns rising and 12 ns trailing edges. Pulse amplitude can be adjusted between 2 and 10 kV. HV pulses are delivered to discharge device through a 50 Ohm coaxial cable (RG213).

Electrical energy dissipated in the plasma is calculated as the difference between emitted and reflected pulses using a back current shunt placed in the middle of a 25-meter cable. The back current shunt consists of 13 resistors of 2.2 Ohm each connected in parallel and soldered at the break in the cable shielding. All electrical signals from the back current shunt were collected using a LeCroy (iX64) oscilloscope.

The discharge device consists of a cylindrical insulated brass electrode covered with a glass cap 1.2 mm thick. The electrode is soldered to the core of the cable and the cable shielding is connected to a copper plate serving as the opposite electrode. A powered electrode is fixed on the translation plate and the inter-electrode gap can be adjusted using a micrometer screw. 

Optical diagnosis: fast imaging of the discharge was performed using an ANDOR iStar DH734 iCCD camera enabling a minimum acquisition gate of 2 ns. Plasma emission imaging is performed through the transparent polymer film with a PtO/Au conductive coating acting as the opposite electrode. Time-resolved emission spectra were measured by ANDOR Shamrock SR-303i spectrometer with a mounted iCCD camera as a detector, in the spectral range 190-900 nm (600 l/mm grating). Plasma emission was collected with an optical fibre placed 1 cm from the discharge in the middle of the gap.

### Plasma treatment of HMEC and Jurkat

The medium in each well was partly removed so that only 200µL remained. For Jurkat cells, attention was paid to avoiding removing the cells that had precipitated in the bottom of the well. The electrode was placed 2mm above the medium surface. The presence of the discharge was confirmed by the emission of a particular sound and a blue light in the dark. After treatment, the medium removed was returned. Experiments were repeated at least three times. The ranges of doses used were determined in preliminary experiments (data not shown). We first implemented a very wide range of doses from 2 to 22500 J/cm^2^ by incrementing the time or the frequency. The scales went up in increments of approximately 100J/cm^2^ initially. The increments were gradually reduced as we reached the appropriate range. We then determined the lowest and highest doses, 9 and 765 J/cm^2^ respectively, which were the most appropriate to detect the whole spectrum of the plasma effects on cells.

### Flow cytometry

The cells were labelled 8 hours or 24 hours after treatment. Jurkat cells were directly collected. HMEC cells were rapidly detached (less than 60 seconds) by Trypsin-EDTA at room temperature. A neutralising solution was added and the cell solutions were collected in cytometry tubes. Cells were counted, washed in Phosphate Buffered Saline solution (PBS) and then re-suspended in 100µl of Annexin-V-buffer-solution (AVBS) with 0.3µl of FITC-Annexin-V solution and 1µl of PI solution (100µg/mL). The cells were incubated in the dark for 15 minutes at room temperature. 400µL of AVBS were added and cytometry analysis was performed.

Cell suspensions were analysed on a Canto II cytometer from Becton Dickinson. Light scatters and fluorescence channels were set at linear and logarithmic gain, respectively.

Analyses were performed on a Forward light scatter (FSC)/Side angle light scatter (SSC) dot plot, on a region containing cells and apoptotic bodies. Apoptosis was analysed on a FITC-Annexin-V/PI dot plot.

### UV, pH and temperature measurements, fluorescence microscopy

For absolute measurements of UV intensity the Andor spectrometer was calibrated with broad band light source (LDLS) EQ-99FC (Energetiq). Then, the discharge was placed 70 cm away from the entrance slit of the monochromator and the emission spectrum in the region of 200-400 nm was accumulated over 500 pulses. The UV meter was obtained from Waldman and placed at the same distance from the plasma as the target during the experiments (skin or cells in culture medium).

For pH measurement of the culture medium after plasma exposure, pH indicator paper (Rota Prolab 35255) and pH probe (Hanna HI 98128) were used. To reduce systematic error, each plasma dose was applied in triplicate on 300 µl of fresh medium. pH readings were taken immediately after the treatment.

We determined plasma temperature using optical emission spectroscopy from the R-branch slope of 337 line (N_2_(C^3^ Π _u_-B^3^ Π _g_, 0-0)) comparing the measured spectrum with the simulated spectrum (Fig.1.g d) [32]. The best fit between two spectral profiles gave the temperature within the error limits.

To measure the electric field inside the plasma discharge we applied a nonintrusive spectroscopic method developed by Paris et al.[33]. It consists in measurement of the intensity ratio of two nitrogen bands corresponding to electronic transitions in nitrogen molecular ions (N_2_^+^(B^2^ Σ _u_^+^-X^2^ Σ _g_^+^, 0-0) and neutral nitrogen molecules (N_2_(C^3^ Π _u_-B^3^ Π _g_, 0-0)). 

We evaluated the presence of intracytoplasmic reactive oxygen species in HMEC by adding 5µM of Cellrox deep Red® to the medium, 1 hour after plasma treatment. As a positive control, cells were incubated with Menadione 100µM instead of plasma treatment. 30 minutes later, the cells were analysed on a fluorescence microscope.

To evaluate electroporation on HMEC, we added Sytox green® to the medium, just before plasma treatment so that the final concentration of the dye was 1µM. The cells were then analysed immediately on a fluorescence microscope.

### Animal model and NTP treatment

Sixteen seven-week-old nude mice were obtained from Iffa Credo (L’Arbresle, France). All the mice were allowed to rest for 1 week before the experiments were conducted. All the mouse studies reported here have been approved by the Animal Housing and Experiment Board of the French government. The NTP technique was applied to each mouse. Each mouse underwent anaesthesia and was given xylazine (10 mg/kg) and ketamine (100 mg/kg) by intra-peritoneal injection. Anaesthesia was ascertained before NTP treatment. Each mouse was treated six times, on three areas delineated on both sides, at increasing frequency or duration. The appropriate range of plasma doses was determined using the results of the cell culture experiments.

Each mouse was then allowed to rest until they were euthanised 5 or 24 hours later. 

### Tissue microscopic analysis

Euthanised mice were immediately dissected, and skin specimens were fixed in 10% formalin and further processed for paraffin embedding. 3 mm–thick paraffin sections were stained with hematoxylin and eosin. 

Some of the specimens were split, one part being fixed in 2% glutaraldehyde in cacodylate buffer and further processed for electron microscopy on Hitachi microscope. Using electronic microscopy, for each sample, on five different fields, 100 cells were counted and classified as normal, necrotic or apoptotic. Results were expressed as median +/- standard error.

### Statistical analyses

Results were expressed as medians. Mann-Whitney’s U-test was used to compare each pair of groups in flow cytometry and electron microscopy data.
